# The pathogenic effector PHYL1_JWB_ interacts with ZjCURT1A to mediate jujube witches' broom‐induced photosynthesis impairment of jujube trees

**DOI:** 10.1111/tpj.71104

**Published:** 2026-09-07

**Authors:** Fangfang Huang, Changfeng Ai, Chong Yang, Jie Feng, Meng Yang, Li Dai, Chaoling Xue, Hongtai Li, Jiaxue Cui, Xiangjie Qu, Jin Zhao, Mengjun Liu, Xuan Zhao, Zhiguo Liu

**Affiliations:** ^1^ College of Horticulture, Hebei Agricultural University Baoding Hebei China; ^2^ National Engineering Research Center for Agriculture in Northern Mountainous Areas Hebei Agricultural University Baoding Hebei China; ^3^ Research Center of Chinese Jujube Hebei Agricultural University Baoding Hebei China; ^4^ Shanxi Sericulture Science Research Institute, Shanxi Agricultural University Yuncheng Shanxi China; ^5^ College of Life Science, Hebei Agricultural University Baoding Hebei China

**Keywords:** jujube witches' broom, PHYL1_JWB_, ZjCURT1A, photosynthesis, chloroplast

## Abstract

Photosynthesis impairment and the accompanying leaf yellowing represent a prominent and physiologically critical symptom of phytoplasma infection. However, the underlying molecular mechanisms remain poorly understood. In this study, we identified the Jujube Witches' Broom pathogenic effector PHYL1_JWB_, which was previously reported to induce phyllody in jujube, as a key inducer of photosynthesis impairment. Overexpression of *PHYL1*_*JWB*_ in sour jujube and *Arabidopsis thaliana* disrupted photosynthesis by causing severe disorganization of chloroplast ultrastructure. PHYL1_JWB_ directly interacts with the integral thylakoid membrane protein ZjCURT1A and promotes its 26S proteasome‐dependent degradation independently of lysine ubiquitination, leading to disruption of thylakoid structure and photosynthetic dysfunction. The conserved two α‐helices of PHYL1_JWB_ and the C‐terminal segment of ZjCURT1A containing the thylakoid membrane curvature‐related CAAD motif are essential for their interaction. Together, these findings reveal that PHYL1_JWB_ impairs photosynthesis by disrupting chloroplast ultrastructural in addition to inducing phyllody, thereby contributing to abnormal plant growth. This work highlights the functional pleiotropy of pathogenic effectors and underscores the need to consider their impacts on multiple physiological processes in the context of phytoplasma pathogenicity.

## Significance Statement

Photosynthesis impairment during phytoplasma infection is mechanistically unclear, yet this work uncovers that the Jujube Witches' Broom pathogenic effector PHYL1_JWB_ directly compromises chloroplast function by promoting 26S proteasome‐dependent degradation of integral thylakoid membrane protein ZjCURT1A, independently of lysine ubiquitination. These findings reveal the pathogenic effector PHYL1_JWB_'s functional pleiotropy beyond phyllody induction, emphasizing that the impacts of pathogenic effectors on multiple physiological processes should be considered in the context of phytoplasma pathogenicity.

## INTRODUCTION

*Candidatus* Phytoplasma comprises a highly diverse genus of bacteria that pose a serious threat to global agriculture and ecosystems. These phloem‐restricted, insect‐transmitted parasites are widely distributed and infect hundreds of plant species, causing severe economic losses (Marcone, 2014; Minato et al., 2014). Spread by leafhoppers, they often trigger devastating, uncontrollable epidemics (Deng et al., 2021; Huang et al., 2021). Phytoplasma infection typically induces a series of characteristic symptoms in plants, including phyllody (the transformation of floral organs into leaf‐like structures) (Ma, Huang, et al., 2024; Ma, Zheng, et al., 2024; MacLean et al., 2011; Maejima et al., 2014; Pracros et al., 2006), witches' broom (excessive shoot proliferation) (Chen et al., 2022; Ma et al., 2023; Wang et al., 2024; Zhou et al., 2021), photosynthesis impairment and leaf yellowing (Hogenhout et al., 2008; Liu et al., 2016). These symptoms are primarily mediated by effector proteins secreted by the phytoplasma, which are unloaded from the phloem into adjacent plant tissues (e.g., shoot and apical meristems) to disrupt normal host development. Several key effector proteins have been identified to date. For example, the homologous effectors SAP54 and PHYL1_OY_ interact with the plant ubiquitin‐binding protein RAD23 and degrades key MADS‐box transcription factors involved in floral development via the ubiquitination pathway, leading to phyllody (Kitazawa et al., 2017, 2022; Maejima et al., 2014). Regarding witches' broom symptoms, effectors SAP05 have been shown to specifically hijack the ubiquitin receptor RPN10 of the host 26S proteasome, degrading SPL and GATA family transcription factors through a unique ubiquitination‐independent mechanism, thereby inducing abnormal shoot proliferation (Liu et al., 2023). The phytoplasma effector PaWB‐SAP54 degrades the SPLa protein via the 26S proteasome, a process dependent on the host protein RPN3. This degradation disrupts the balance of auxin, gibberellin, and cytokinin in *Paulownia* plants, ultimately inducing the characteristic witches'‐broom symptoms (Cao et al., 2021).

Photosynthesis impairment and the accompanying leaf yellowing represent a particularly prominent and physiologically critical symptom caused by phytoplasma infection (Gupta et al., 2023; Liu et al., 2016; Mittelberger et al., 2017; Teixeira et al., 2020; Wei et al., 2022). This process involves a systemic collapse of the photosynthetic apparatus, encompassing both structural integrity and functional capacity (Xue et al., 2018). Specifically, it is characterized by disrupted chloroplast development, impaired biosynthesis of photosynthetic pigments, damage to chloroplast ultrastructure, and inhibition of photosynthetic electron transport and key enzymes involved in carbon assimilation, all of which collectively contribute to a substantial decline in photosynthetic efficiency. Dysfunction of the photosynthetic machinery reduces carbohydrate synthesis, leading to energy and nutrient deficiencies, which ultimately result in growth arrest, premature senescence, and plant death (Liu et al., 2016; Xue et al., 2018). Nevertheless, the molecular mechanisms underlying photosynthetic impairment induced by phytoplasma infections in plants remain poorly understood.

Chinese jujube (*Ziziphus jujuba* Mill.), an economically important fruit tree native to China renowned for its nutritional value and cultural significance (Huang et al., 2026; Lin et al., 2025; Pan et al., 2025; Wang et al., 2025, 2026; Yang et al., 2023), is severely affected by Jujube Witches' Broom (JWB), a typical phytoplasma disease. JWB is one of the most destructive constraints to jujube production, often leading to severe yield losses and orchard decline (Ai et al., 2026; Liu et al., 2020; Ma, Huang, et al., 2024; Ma, Zheng, et al., 2024; Xue et al., 2020). As a hallmark symptom of JWB, photosynthesis impairment directly affects the normal growth and development of jujube trees (Liu et al., 2016; Xue et al., 2024). Chloroplasts are the primary sites of photosynthesis and rely on structural integrity for efficient light reactions. This integrity particularly depends on the proper expression and localization of protein complexes on the thylakoid membranes. Studies have shown that phytoplasma infection disrupts thylakoid membrane structure and alters the expression profile of membrane proteins, thereby reducing photosynthetic efficiency (Li et al., 2021; Xue et al., 2018). This is manifested as decreased chlorophyll content, inhibited electron transport, and reduced photosynthetic products, ultimately leading to leaf yellowing and tree decline (Liu et al., 2016; Xue et al., 2018).

CURVATURE THYLAKOID1 (CURT1) protein family is a key and conserved plant‐specific protein group that regulates thylakoid morphology in chloroplasts. In *Arabidopsis thaliana*, the CURT1 protein family consists of four isoforms (CURT1A, B, C, and D). Among these, CURT1A is the most abundant subunit in chloroplasts. These proteins oligomerize to form large complexes that localize to the margins of grana stacks as structural scaffolds. By inducing and maintaining negative curvature of the thylakoid membrane in these regions, they precisely regulate grana diameter and lamella flatness (Armbruster et al., 2013; Trotta et al., 2025). Membrane bending is a prerequisite for the formation of typical grana stacks, and CURT1 proteins facilitate this process in a dose‐dependent manner. This regulatory role in thylakoid membrane morphology is crucial for the proper assembly and function of the photosynthetic apparatus. In the *curt1abcd* quadruple mutant of Arabidopsis, thylakoid architecture is severely disrupted: grana stacking is significantly reduced, with most grana consisting of only single or few membrane layers, leading to a disorganized thylakoid network. This structural defect directly impairs photosynthetic performance, particularly by disrupting the spatial arrangement of Photosystem II (PSII) and its light‐harvesting complexes, thereby reducing the efficiency of light capture and electron transport. In contrast, overexpression of *CURT1* leads to altered grana stack morphology, characterized by taller, narrower stacks with increased membrane layers and more developed margins (Pribil et al., 2018). The plasticity mediated by CURT1 not only underlies the dynamic reorganization of thylakoid ultrastructure but is also essential for maintaining photosynthetic efficiency and overall plant adaptability. Studies have shown that under unfavorable growth conditions, any destabilizing alteration in thylakoid ultrastructure can significantly negatively affect plant growth (Armbruster et al., 2013; Pribil et al., 2018; Trotta et al., 2025).

In this study, we found that the JWB pathogenic effector PHYL1_JWB_, which was previously reported to induce phyllody in jujube (Xue et al., 2024), was highly expressed in yellowing leaves of JWB‐infected jujube trees. Overexpression of *PHYL1*_*JWB*_ in wild jujube and *A. thaliana* impaired photosynthesis by altering chloroplast ultrastructure. Further investigation revealed that PHYL1_JWB_ interacted with the integral thylakoid membrane protein ZjCURT1A in chloroplasts and mediated its degradation via 26S proteasome but was independent of substrate lysine ubiquitination. Our findings demonstrated that besides inducing phyllody, PHYL1_JWB_ also directly impaired photosynthesis and reduced photosynthetic efficiency by disrupting chloroplast ultrastructure. This discovery expands the theoretical framework of phytoplasma pathogenicity and provides a crucial scientific basis for developing comprehensive strategies for JWB prevention and control.

## RESULTS

### Phytoplasma impairs the photosynthetic system and reduces photosynthetic capacity in jujube plants with JWB

**Figure 1:**
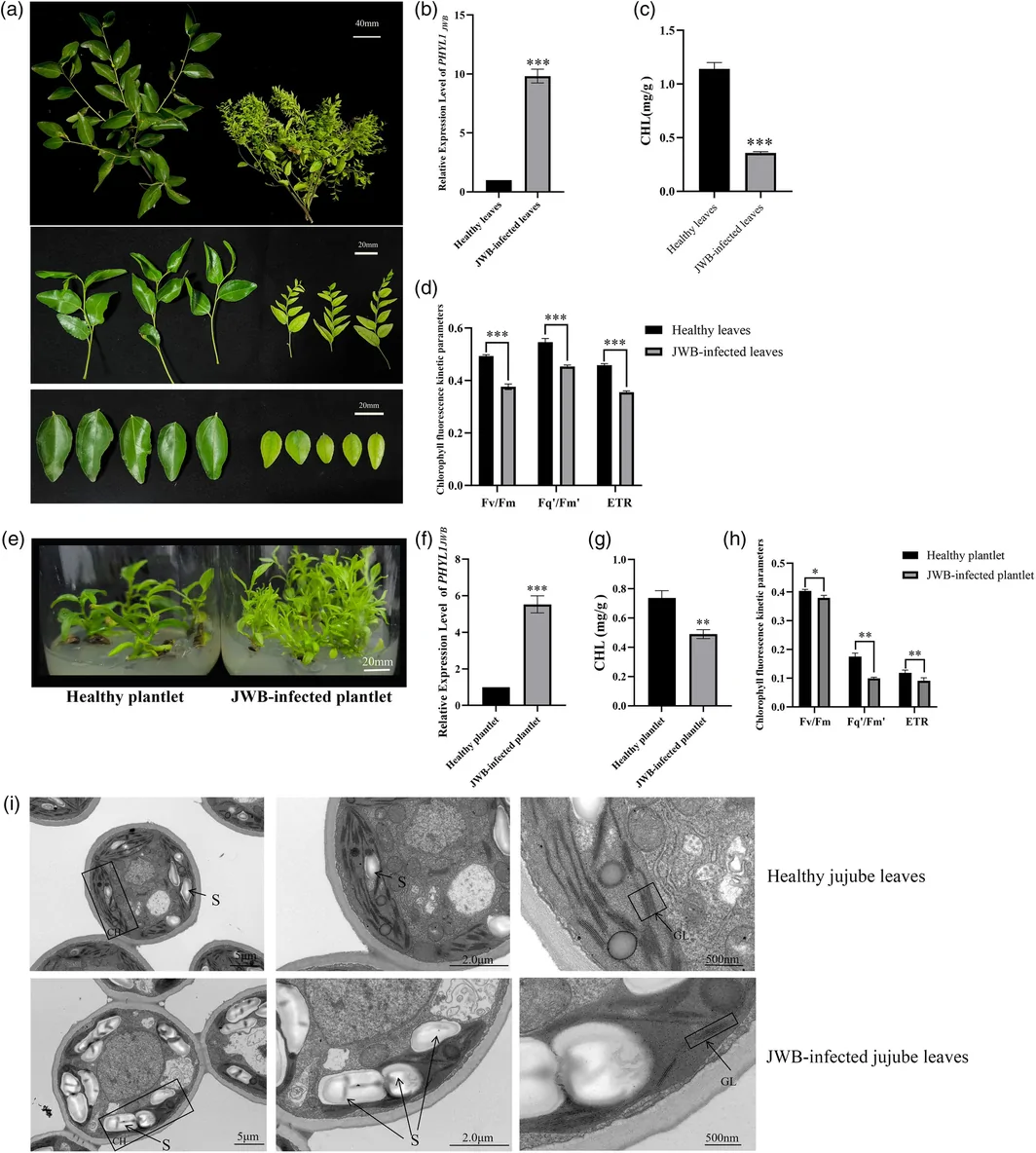
Jujube witches' broom (JWB) leads to damage in the photosynthetic system and a decline in photosynthetic capacity of jujube plants. (a) Phenotypic images of healthy jujube leaves (left) and JWB‐infected jujube leaves (right) in field‐grown trees. (b) The phytoplasma effector *PHYL1*_*JWB*_ was expressed significantly higher in JWB‐infected jujube leaves than in healthy ones. (c) Chlorophyll content in JWB‐infected jujube leaves was significantly reduced compared to that in healthy jujube leaves. (d) Chlorophyll fluorescence kinetic parameters in JWB‐infected jujube leaves and healthy jujube leaves. (e) Phenotypic images of healthy jujube plantlet and JWB‐infected jujube plantlet. (f) Relative expression of *PHYL1*_*JWB*_ in healthy jujube plantlet and JWB‐infected jujube plantlet. (g) Chlorophyll content in JWB‐infected jujube plantlet was significantly reduced compared to that in healthy jujube plantlet. (h) Chlorophyll fluorescence kinetic parameters in healthy jujube plantlet and JWB‐infected jujube plantlet. (i) Transmission electron microscopy (TEM) micrographs of ultrathin sections of leaves from healthy jujube and JWB‐infected jujube. CH, chloroplast; GL, grana lamella; S, starch granule. Significant differences were based on Student's *t*‐test:*P < 0.05, **P < 0.01 and ***P < 0.001.

Upon infection with JWB, jujube trees exhibited typical foliar symptoms, including reduced leaf area and yellowing. These symptoms were observed both in field‐grown trees and in tissue‐cultured plantlets (Figure 1a,e). Measurement of chlorophyll content (CHL) in the leaves of JWB‐infected trees revealed a significant decrease compared to the leaves of healthy jujube plants (Figure 1c,g). Furthermore, analysis of chlorophyll kinetics‐related parameters showed that the average values of *F*_v_/*F*_m_ (maximum photochemical quantum yield), *F*_q_'/*F*_m_' (actual photochemical efficiency), and ETR (electron transport rate) in the infected leaves were significantly lower than those in healthy jujube leaves (Figure 1d,h), indicating that JWB leads to damage in the photosynthetic system and a decline in photosynthetic capacity.

To investigate whether the phytoplasma effector PHYL1_JWB_ contributes to the impairment of photosynthesis, we examined its expression in JWB‐infected leaves relative to healthy controls, in both field‐grown trees and tissue‐cultured plantlets. We found that the expression of *PHYL1*_*JWB*_ was significantly up‐regulated in JWB‐infected plants (Figure 1b,f), implicating its potential role in mediating the damage to the photosynthetic system caused by JWB.

To determine the underlying cause of photosynthetic impairment in leaves affected by JWB, we examined chloroplast ultrastructure in field‐collected leaves from healthy and JWB‐infected trees using transmission electron microscopy (TEM). In healthy jujube leaves, the chloroplasts exhibited normal development, with few and morphologically typical starch granules. The grana lamellae, which are formed by the folding of thylakoid membranes and serve as the key sites for photosynthesis, were intact and regularly arranged (Figure 1i). In contrast, JWB‐infected leaf cells exhibited a greater number of enlarged starch granules, which compressed the photosynthetically active regions of the chloroplasts. Moreover, both the number of grana lamellae and the extent of their stacking were markedly reduced (Figure 1i). These findings indicated that the disruption of chloroplast ultrastructure induced by JWB infection was a key factor responsible for the reduced chlorophyll content and the consequent decrease in photosynthetic activity.

### The phytoplasma effector PHYL1_JWB_ impairs photosynthesis by altering chloroplast ultrastructure

**Figure 2:**
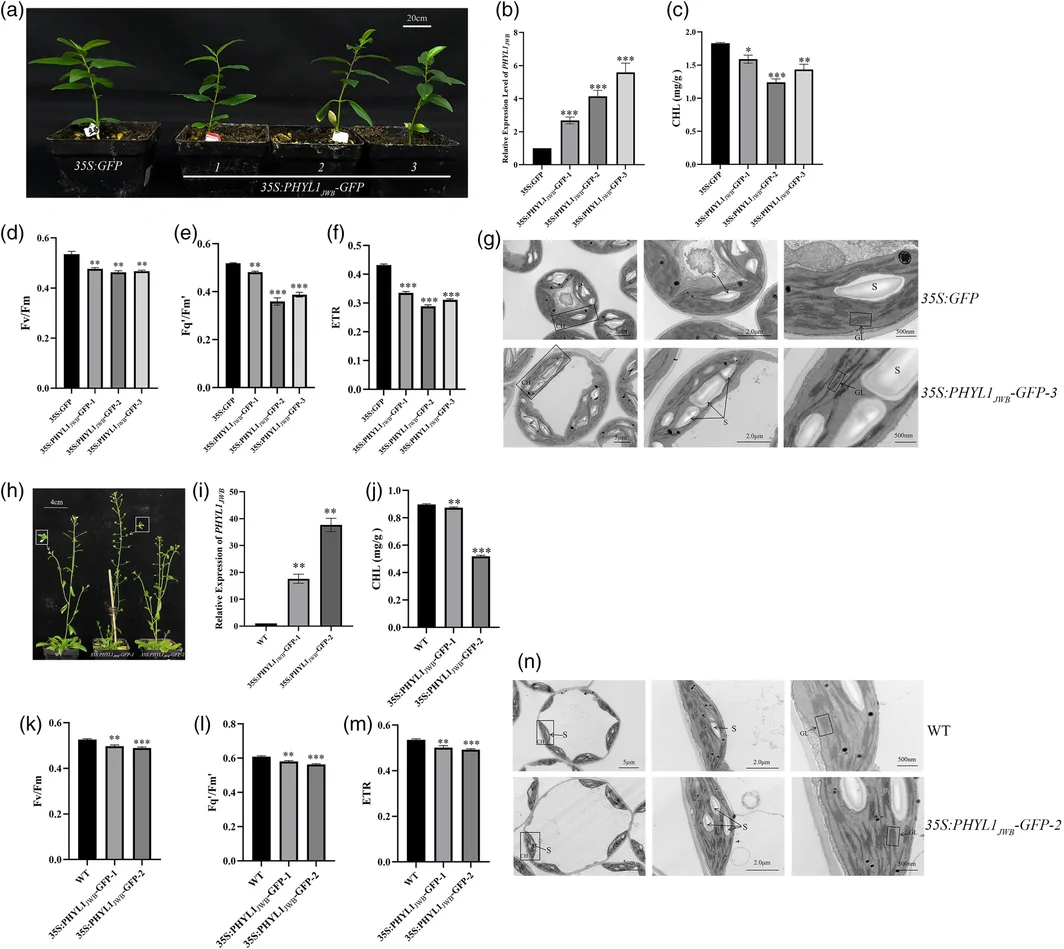
Overexpression of phytoplasma effector *PHYL1*_*JWB*_ impairs photosynthesis by altering chloroplast ultrastructure in both jujube and Arabidopsis seedlings. (a) Phenotypic images of *35S:GFP* (control) and *35S:PHYL1*_*JWB*_ *‐GFP* overexpressing plants of sour jujube seedlings. (b) The relative expression of phytoplasma effector *PHYL1*_*JWB*_ in *35S:GFP* and *35S:PHYL1*_*JWB*_ *‐GFP* overexpressing plants of sour jujube seedlings. (c) Chlorophyll content in *PHYL1*_*JWB*_ *‐GFP* overexpressing plants of sour jujube seedlings was significantly reduced compared to that in *35S:GFP* plants. (d–f) The value of *F*_v_/*F*_m_, *F*_q_'/*F*_m_' and electron transport rate (ETR) in *35S:GFP* and *35S:PHYL1*_*JWB*_ *‐GFP* overexpressing plants of sour jujube seedlings. (g) Transmission electron microscopy (TEM) micrographs of ultrathin sections of leaves from *35S:GFP* and *35S:PHYL1*_*JWB*_ *‐GFP* overexpressing plants of sour jujube seedlings. CH, chloroplast; GL, grana lamella; S, starch granule. (h) Phenotypic images of wild‐type (WT) and *35S:PHYL1*_*JWB*_ *‐GFP* overexpressing plants of Arabidopsis. The flowers in the white box showed that WT had normal floral structures, whereas *PHYL1*_*JWB*_ overexpression lines exhibited a ‘flower‐to‐leaf’ structural transformation. (i) The relative expression of phytoplasma effector *PHYL1*_*JWB*_ in WT and *35S:PHYL1*_*JWB*_ *‐GFP* overexpressing plants of Arabidopsis. (j) Chlorophyll content in *PHYL1*_*JWB*_ *‐GFP* overexpressing plants of Arabidopsis was significantly reduced compared to that in WT. (k–m) The value of *F*_v_/*F*_m_, *F*_q_'/*F*_m_' and ETR in *35S:GFP* and *35S:PHYL1*_*JWB*_ *‐GFP* overexpressing plants of Arabidopsis. (n) TEM micrographs of ultrathin sections of leaves from WT and *35S:PHYL1*_*JWB*_ *‐GFP* overexpressing plants of Arabidopsis. CH, chloroplast; GL, grana lamella; S, starch granule. Significant differences were based on Student's *t*‐test:*P < 0.05, **P < 0.01 and ***P < 0.001.

To determine whether the phytoplasma effector PHYL1_JWB_ is responsible for the photosynthetic damage induced by JWB, we generated *35S:PHYL1*_*JWB*_ *‐GFP* overexpressing plants via transient transformation of sour jujube seedlings (Figure 2a). Thirty days after transfection, the leaves of *35S:PHYL1*_*JWB*_ *‐GFP* overexpression seedlings showed significantly reduced chlorophyll content compared to the 3*5S:GFP* control (Figure 2a–c). Furthermore, the average values of chlorophyll kinetics‐related parameters, including *F*_v_/*F*_m_, *F*_q_'/*F*_m_', and ETR, were significantly lower in the transgenic seedlings (Figure 2d–f), confirming that *PHYL1*_*JWB*_ overexpression directly caused photosynthetic system damage and a decline in photosynthetic performance.

Ultrastructural observation of chloroplasts in jujube seedlings overexpressing *35S:PHYL1*_*JWB*_ *‐GFP* and those expressing the empty vector control (3*5S:GFP*) revealed that, compared with the control, the transgenic seedlings overexpressing *PHYL1*_*JWB*_ exhibited more numerous and enlarged starch granules within the chloroplasts, along with a significant reduction in both the number of grana lamellae and their stacking layers (Figure 2g). These findings indicated that expression of the phytoplasma effector protein *PHYL1*_*JWB*_ disrupted chloroplast ultrastructure in leaf cells, thereby reducing chlorophyll content and impairing subsequent photosynthetic performance.

We also generated *PHYL1*_*JWB*_‐overexpressing lines in *A. thaliana*. The *PHYL1*_*JWB*_‐overexpressing plants not only exhibited the previously reported phyllody phenotype but also recapitulated the significant reduction in chlorophyll content observed in jujube (Figure 2h–j). Moreover, they showed significantly decreased average values in chlorophyll kinetics‐related parameters (*F*_v_/*F*_m_, *F*_q_'/*F*_m_', and ETR) compared to the wild‐type (WT) (Figure 2k–m). In addition, ultrastructural observation of chloroplasts in leaves of *A. thaliana* overexpressing *35S:PHYL1*_*JWB*_ *‐GFP* revealed that, compared to WT plants, the overexpression lines accumulated more numerous and enlarged starch granules within the chloroplasts, accompanied by a significant reduction in both the number of grana lamellae and their stacking layers (Figure 2n). These data collectively provided compelling evidence that PHYL1_JWB_ was sufficient to disrupt the photosynthetic system, diminish photosynthetic capacity by altering chloroplast ultrastructure.

### PHYL1_JWB_ interacts with the integral thylakoid membrane protein CURVATURE THYLAKOID 1A on the chloroplast

**Figure 3:**
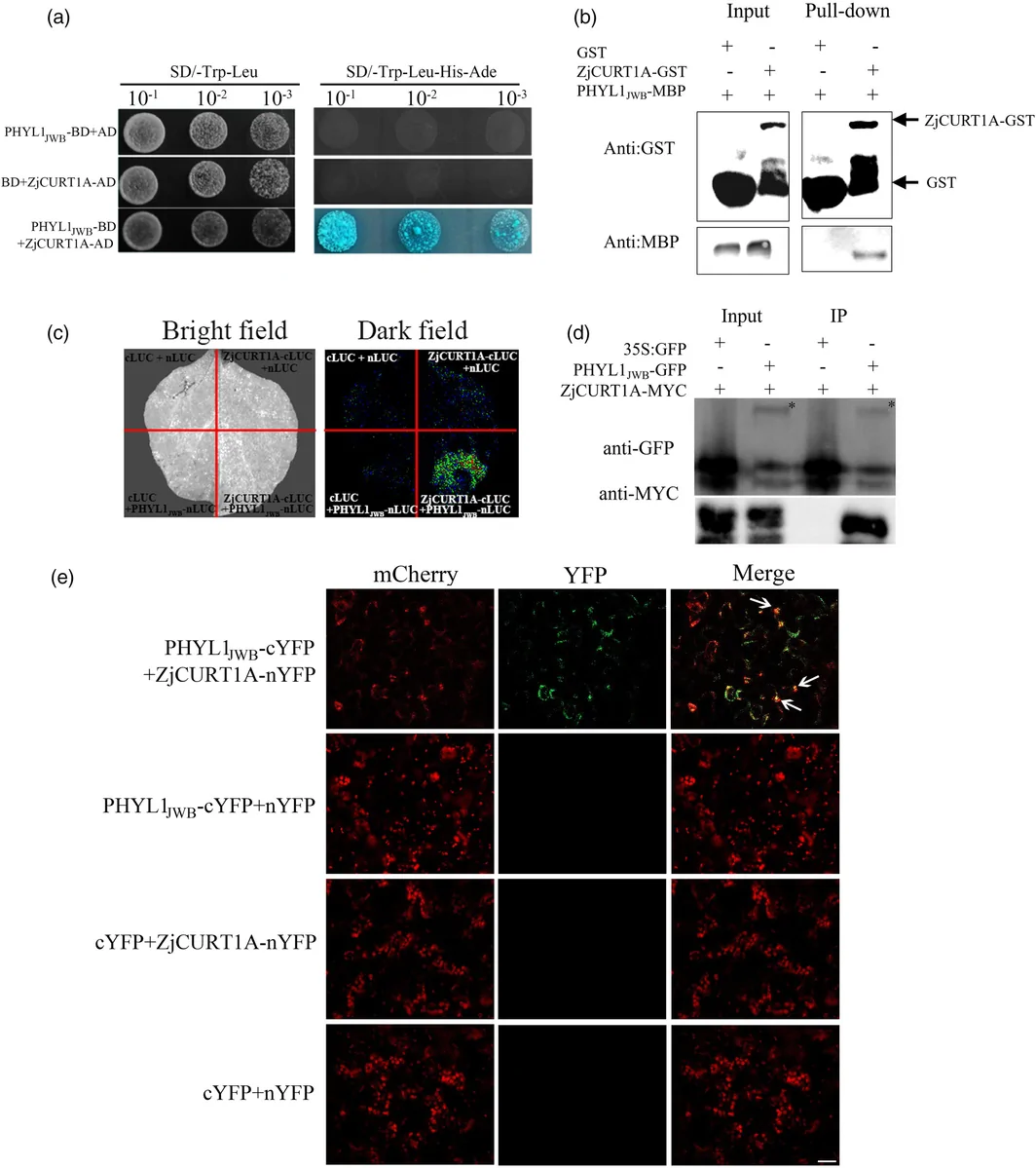
PHYL1_JWB_ physically interacts with ZjCURT1A. (a) The yeast two‐hybrid assay showed that PHYL1_JWB_ interacted with ZjCURT1A but not with the negative controls. (b) *In vitro* pull‐down assays of PHYL1_JWB_ with ZjCURT1A. Equal amounts of affinity‐purified PHYL1_JWB_‐MBP were incubated with ZjCURT1A‐GST and GST. Pull‐down proteins were subjected to immunoblotting with anti‐MBP antibodies. PHYL1_JWB_‐MBP was specifically pulled down by ZjCURT1A‐GST but not the GST control. (c) Luciferase complementation imaging (LCI) assay in *Nicotiana benthamiana*. Signals were only detected with the co‐transformation of *PHYL1*_*JWB*_ and *ZjCURT1A*. Empty vectors were used as negative controls. (d) Co‐immunoprecipitation (Co‐IP) assay showed an interaction between PHYL1_JWB_ and ZjCURT1A *in vivo*. Following incubation of total protein extracts with anti‐GFP agarose, immunoblotting with an anti‐MYC antibody revealed that ZjCURT1A co‐precipitated with PHYL1_JWB_‐GFP, but not with the GFP control. * indicated PHYL1_JWB_‐GFP. (e) Bimolecular fluorescence complementation (BiFC) assay in *N. benthamiana*. Plasmids of the indicated gene sets were transiently co‐expressed in tobacco leaves. A reconstituted YFP signal was observed only in cells co‐infiltrated with *PHYL1*_*JWB*_ *‐cYFP* and *ZjCURT1A‐nYFP*, whereas control combinations with empty vectors showed no fluorescence. *pBI121‐35S‐Chlo‐mCherry* was used as a chloroplast marker. Bars, 50 μm.

Using a yeast two‐hybrid (Y2H) screening assay, an interacting protein of PHYL1_JWB_ was identified, namely the integral thylakoid membrane protein ZjCURT1A, which is required for proper grana stack curvature. To confirm this interaction, a one‐to‐one Y2H assay was performed. As shown in Figure 3a, only yeast co‐expressing both PHYL1_JWB_ and ZjCURT1A grew on selective medium, whereas control strains expressing AD with PHYL1_JWB_‐BD or BD with ZjCURT1A‐AD did not, indicating a specific physical interaction between PHYL1_JWB_ and ZjCURT1A. We further validated this interaction using an *in vitro* pull‐down assay with GST‐ZjCURT1A as bait. The results demonstrated that PHYL1_JWB_‐MBP was specifically pulled down and detected with an anti‐MBP antibody, while the GST control showed no binding (Figure 3b). Subsequently, to investigate the interaction between PHYL1_JWB_ and ZjCURT1A *in vivo*, we performed luciferase complementation imaging (LCI) and co‐immunoprecipitation (Co‐IP) assay in tobacco. Luciferase activity was detected only when PHYL1_JWB_ and ZjCURT1A were co‐expressed in tobacco leaves (Figure 3c). In the Co‐IP assay, total proteins were extracted and incubated with anti‐GFP agarose beads. The precipitated proteins were then detected using an anti‐MYC antibody. As shown in Figure 3d, ZjCURT1A specifically co‐immunoprecipitated with PHYL1_JWB_‐GFP, but not with GFP alone. In addition, bimolecular fluorescence complementation (BiFC) assays in plant cells showed that a YFP signal was observed in chloroplasts when *35S:PHYL1*_*JWB*_ *‐cYFP* was co‐infiltrated with *35S:ZjCURT1A‐nYFP*, which was co‐localized with a chloroplast marker, but not in any of the control combinations (Figure 3d). These findings confirmed that PHYL1_JWB_ and ZjCURT1A interacted with each other specifically within chloroplasts.

### Alterations in *ZjCURT1A* expression levels modify the chloroplast ultrastructure

**Figure 4:**
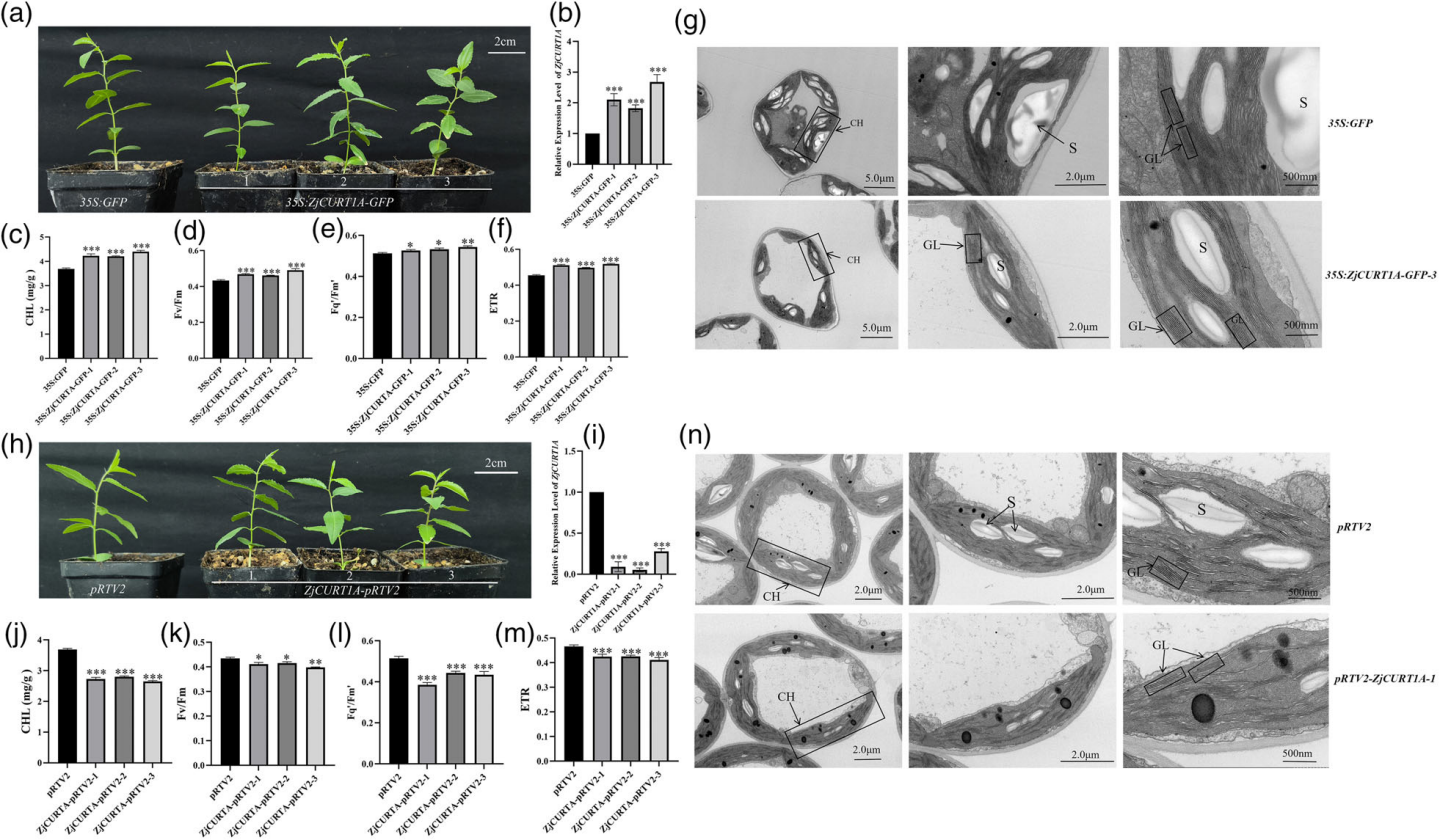
Function analysis of the chloroplast thylakoid membrane protein ZjCURT1A. (a) Phenotypic images of *35S:GFP* (empty vector, control) and *35S:ZjCURT1A* overexpressing plants of sour jujube seedlings. (b) The relative expression of *ZjCURT1A* in *35S:GFP* and *35S:ZjCURT1A‐GFP* overexpressing plants of sour jujube seedlings. (c) Chlorophyll contents in *35S:ZjCURT1A‐GFP* overexpressing plants of sour jujube seedlings were significantly increased compared to that in *35S:GFP* plants. (d–f) The values of *F*_v_/*F*_m_, *F*_q_'/*F*_m_' and electron transport rate (ETR) in *35S:GFP* and *35S:ZjCURT1A‐GFP*overexpressing plants of sour jujube seedlings. (g) Transmission electron microscopy (TEM) micrographs of ultrathin sections of leaves from *35S:GFP* and *35S:ZjCURT1A‐GFP* overexpressing plants of sour jujube seedlings. CH, chloroplast; GL, grana lamella; S, starch granule. (h) Phenotypic images of *pTV2* (empty vector, control) and *pRTV2‐ZjCURT1A* silencing plants of sour jujube seedlings. (i) The relative expression of *ZjCURT1A* in control and *pRTV2‐ZjCURT1A* silencing plants of sour jujube seedlings. (j) Chlorophyll contents in *pRTV2‐ZjCURT1A* silencing plants of sour jujube seedlings were significantly reduced compared to that in control. (k–m) The values of *F*_v_/*F*_m_, *F*_q_'/*F*_m_' and ETR in control and *pRTV2‐ZjCURT1A* silencing plants of sour jujube seedlings. (n) TEM micrographs of ultrathin sections of leaves from control and *pRTV2‐ZjCURT1A* silencing plants of sour jujube seedlings. CH, chloroplast; GL, grana lamella; S, starch granule. Significant differences were based on Student's *t*‐test:*P < 0.05, **P < 0.01 and ***P < 0.001.

To investigate the function of the chloroplast thylakoid membrane protein ZjCURT1A, it was transiently overexpressed in jujube seedlings and obtained *35S:ZjCURT1A‐GFP* overexpressing lines (Figure 4a,b). Meanwhile, virus‐induced gene silencing (VIGS) was employed to knock down *ZjCURT1A* expression in jujube seedlings and obtained *pRTV2‐ZjCURT1A* lines (Figure 4h,i). Chlorophyll content measurements revealed that *pRTV2‐ZjCURT1A* plants exhibited reduced chlorophyll levels, whereas *35S:ZjCURT1A‐GFP* overexpressing plants showed increased chlorophyll content (Figure 4c,j). Analysis of chlorophyll kinetics‐related parameters demonstrated that the average values of *F*_v_/*F*_m_, *F*_q_'/*F*_m_', and ETR were significantly higher in *ZjCURT1A*‐overexpressing plants than in control plants (Figure 4d–f). In contrast, these parameters were significantly lower in *ZjCURT1A* silenced plants compared to the control (Figure 4k–m). These results indicated that the expression of *ZjCURT1A* was closely associated with the regulation of the photosynthetic system in plants.

To investigate whether the expression of *ZjCURT1A* affects chloroplast ultrastructure, we conducted anatomical and TEM observations on leaves of *35S:ZjCURT1A‐GFP* overexpressing, *pRTV2‐ZjCURT1A* silencing plants and their respective empty vector transgenic control plants. The results revealed that, compared with the control *35S:GFP* plants, the chloroplasts in mesophyll cells of *ZjCURT1A*‐overexpressing seedlings exhibited enlarged volume, fewer starch grains, increased stromal lamellae, and extended thylakoid membrane stacking area (Figure 4g). These structural changes enhanced light‐harvesting capacity and improved electron transport efficiency. In contrast, compared with the control, chloroplasts in *ZjCURT1A*‐silenced mesophyll cells accumulated more starch granules, contained fewer grana, and exhibited reduced stacking of grana lamellae (Figure 4n). These alterations collectively resulted in decreased light capture, impaired electron transport, and reduced net photosynthetic rate. Therefore, changes in *ZjCURT1A* expression levels led to modifications in the ultrastructure of chloroplast thylakoids, thereby influencing photosynthetic performance.

### PHYL1_JWB_ regulates the degradation of ZjCURT1A via the 26S proteasome, but independent of substrate ubiquitination

**Figure 5:**
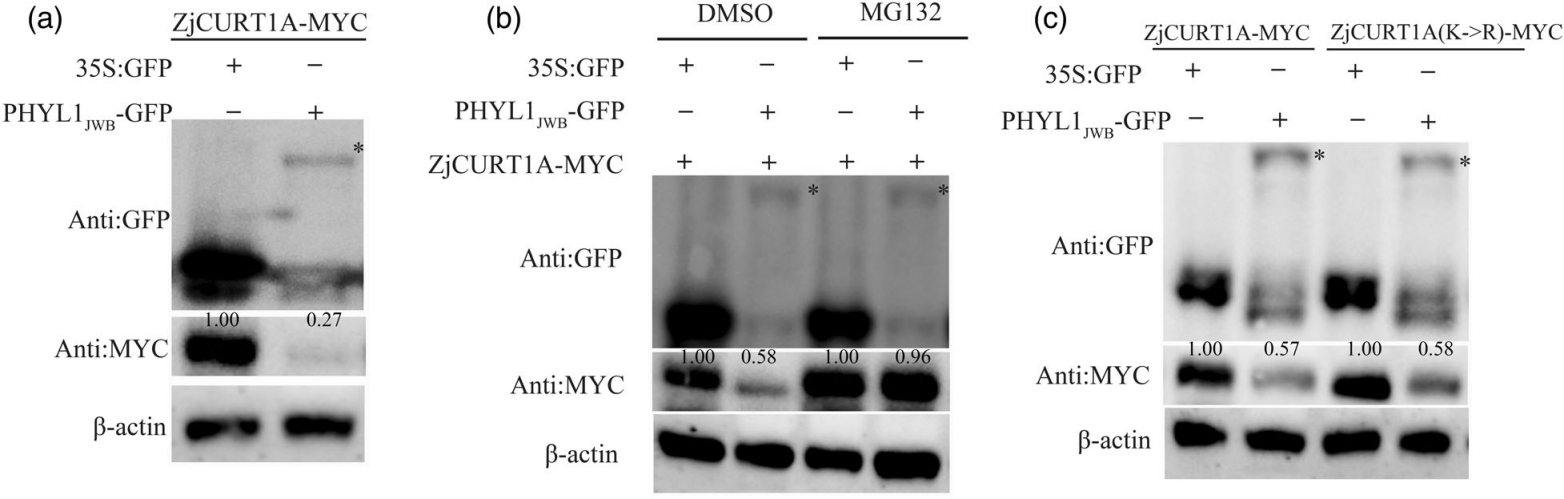
PHYL1_JWB_ regulates the degradation of ZjCURT1A via the 26S proteasome but independent of substrate ubiquitination. (a) Western blot analysis showed that the protein level of ZjCURT1A‐MYC was significantly degraded when PHYL1_JWB_‐GFP was expressed. * indicated PHYL1_JWB_‐GFP. (b) Western blot analysis showed that the specific 26S proteasome inhibitor MG132 suppressed the PHYL1_JWB_‐GFP‐induced degradation of the ZjCURT1A‐MYC protein. DMSO, dimethylsulfoxide. (c) Western blot analysis showed that the PHYL1_JWB_‐GFP‐induced degradation of the ZjCURT1A‐MYC protein did not depend on ubiquitination of the ZjCURT1A‐MYC protein. K → R, all lysine residues were replaced by arginine residues in ZjCURT1A proteins. Band intensities were measured using ImageJ software and normalized against the β‐Actin loading control.

Previous studies reported that PHYL1_JWB_ interacts with the 26S proteasome subunit RAD23C to mediate the degradation of MADS‐box proteins, thereby regulating the phytoplasma‐induced leaf‐flower transformation in jujube (Xue et al., 2024). This led us to investigate whether the interaction between PHYL1_JWB_ and ZjCURT1A affects the protein stability of ZjCURT1A. We co‐expressed 35S:ZjCURT1A‐MYC and 35S:PHYL1_JWB_‐GFP in tobacco leaves. The results showed that compared with the control, the protein level of ZjCURT1A was significantly reduced when PHYL1_JWB_ was expressed (Figure 5a), indicating that PHYL1_JWB_ promoted the destabilization of ZjCURT1A.

To determine whether PHYL1_JWB_‐induced destabilization of ZjCURT1A depends on the 26S proteasome, we treated tobacco leaves co‐expressing PHYL1_JWB_ and ZjCURT1A with MG132, a specific 26S proteasome inhibitor, and measured the protein level of ZjCURT1A. We found that MG132 treatment suppressed the PHYL1_JWB_‐mediated decrease in ZjCURT1A protein abundance compared to the DMSO control (Figure 5b). These results suggest that ZjCURT1A was destabilized by PHYL1_JWB_ through the host 26S proteasome pathway.

To investigate whether PHYL1_JWB_‐mediated degradation of ZjCURT1A depends on the ubiquitination of ZjCURT1A itself, we substituted all lysine residues in ZjCURT1A with arginines and examined the protein levels in the presence of PHYL1_JWB_. The results showed that, when PHYL1_JWB_ was expressed, both the WT and mutant forms of ZjCURT1A were significantly reduced compared to the 35S:GFP control. These findings suggested that lysine‐based ubiquitination of the substrate was not required for PHYL1_JWB_‐mediated degradation of ZjCURT1A, indicating that PHYL1_JWB_ promoted ZjCURT1A degradation through a ubiquitination‐independent mechanism. This mode of action is consistent with the previously reported PHYL1_JWB_‐mediated degradation of MADS‐box proteins (Xue et al., 2024).

### The two α‐helical helixes domains of PHYL1_JWB_ protein are essential for its interaction with ZjCURT1A

**Figure 6:**
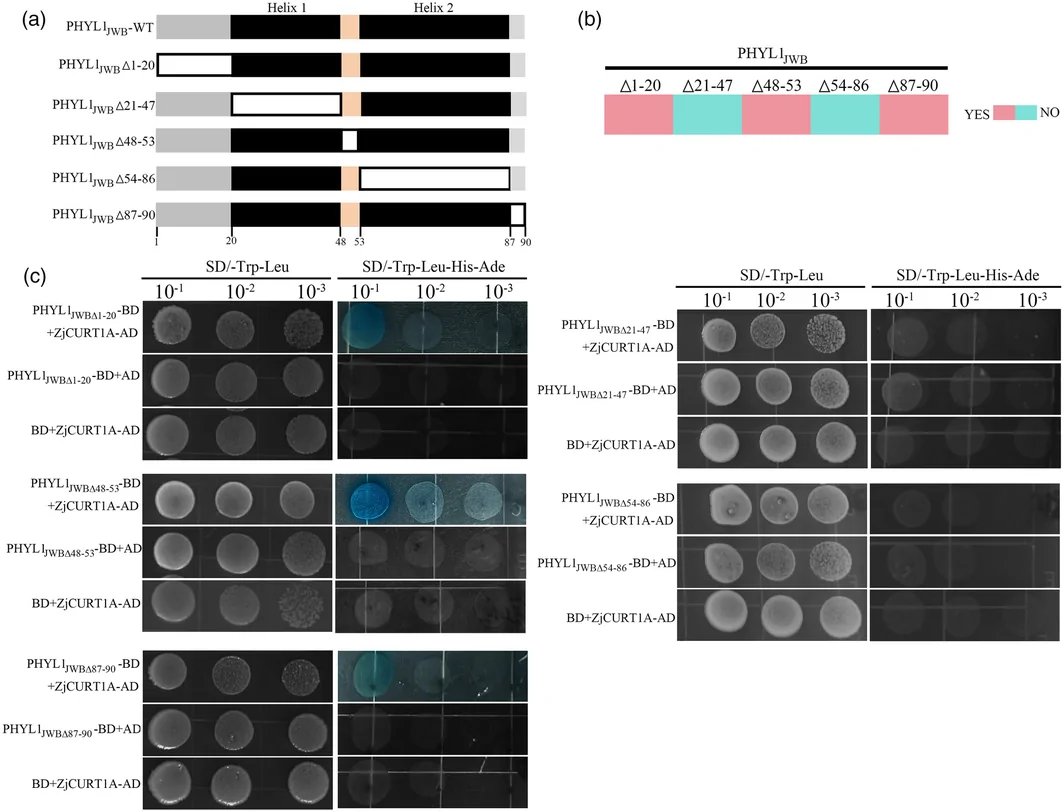
The loss of the two α‐helix domains in PHYL1_JWB_ abolishes its interaction with ZjCURT1A. (a) Illustration of the PHYL1_JWB_ mutants generated via deletion of specific amino acid fragments. The construct PHYL1_JWB_‐WT represents the wild‐type sequence without its signal peptide. Successive deletions are designated as follows: PHYL1_JWB_Δ1–20 (first random coil), PHYL1_JWB_Δ21–47 (first α‐helix), PHYL1_JWB_Δ48–53 (second random coil), PHYL1_JWB_Δ54–86 (second α‐helix), and PHYL1_JWB_Δ87–90 (final random fragment). Deleted regions are indicated by blank boxes. (b) Summarization of yeast two‐hybrid (Y2H) assay results for the interaction between PHYL1_JWB_ mutants and ZjCURT1A. In the schematic, a pink box denotes a positive interaction, whereas a green box indicates no interaction. (c) The results of Y2H assays showing the interaction between ZjCURT1A and the truncated PHYL1_JWB_ mutants.

According to previous studies, the PHYL1_JWB_ protein consists of two α‐helices, α1 (amino acids 21–47) and α2 (amino acids 54–86), and two random coil regions (amino acids 1–20 and 48–53), with the two helices being separated by the latter coil (Figure 6a). To elucidate the structural basis of the PHYL1_JWB_–ZjCURT1A interaction, we generated a series of truncated variants of PHYL1_JWB_ (Figure 6a). Y2H assays revealed that yeast colonies co‐expressing either PHYL1_JWB_Δ21–47 or PHYL1_JWB_Δ54–86 with ZjCURT1A failed to grow on the selective medium, whereas all other truncated PHYL1_JWB_ variants co‐expressed with ZjCURT1A grew normally on the same medium (Figure 6b,c). These results demonstrated that both the α1 and α2 helices were indispensable for the interaction between PHYL1_JWB_ and ZjCURT1A.

### Structural analysis of the essential sites for PHYL1_JWB_ interaction with ZjCURT1A

**Figure 7:**
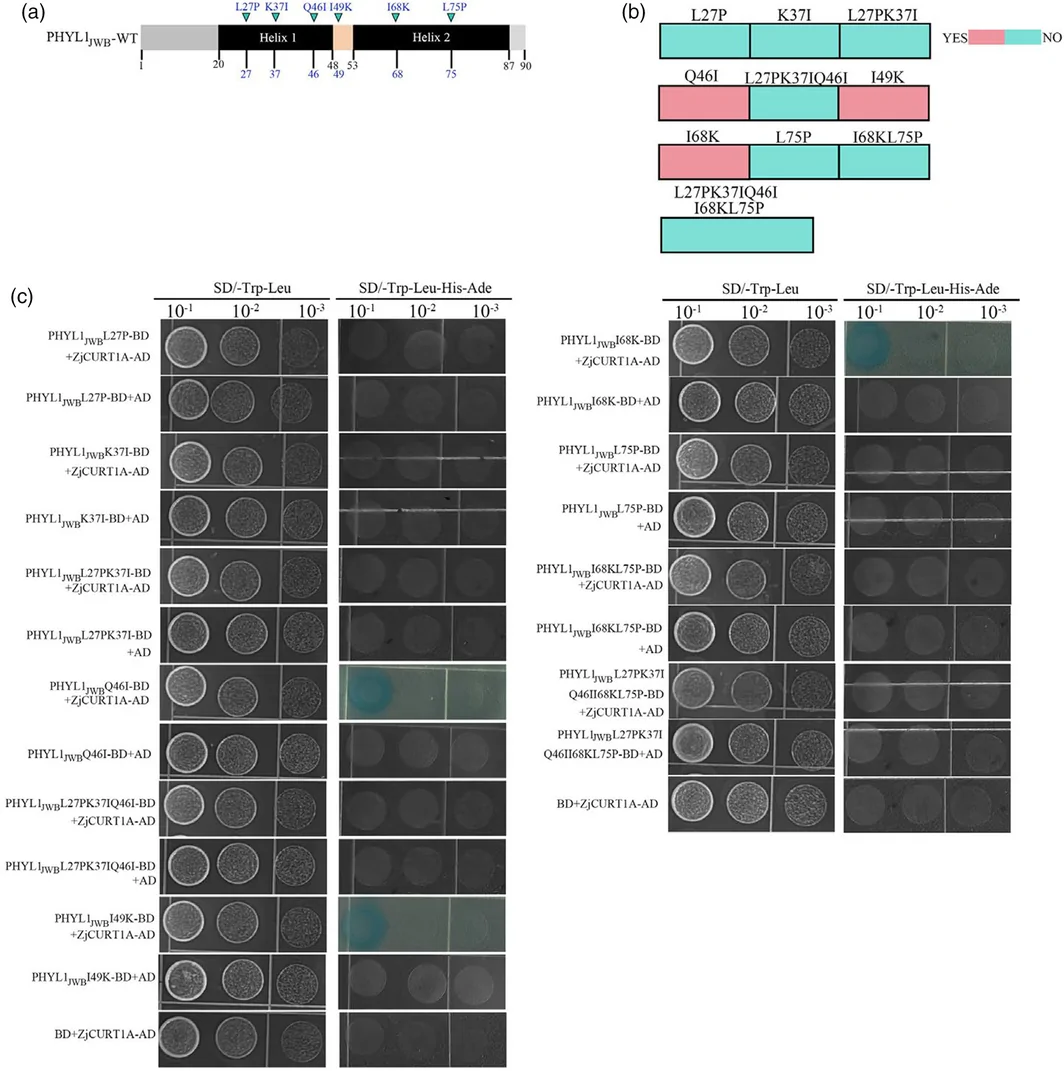
Yeast two‐hybrid (Y2H) assays of the interaction between PHYL1_JWB_ mutants generated by amino acid substitution with ZjCURT1A. (a) Schematic of the PHYL1_JWB_ mutants generated by amino acid substitution. Numbers indicate the amino acid positions (starting from the initiation codon). (b) Summarization of Y2H assay results for the interaction between PHYL1_JWB_ mutants and ZjCURT1A. In the schematic, a pink box denotes a positive interaction, whereas a green box indicates no interaction. (c) The results of Y2H assays showing the interaction between ZjCURT1A and the PHYL1_JWB_ mutants.

To identify the specific residues within the two α‐helices of PHYL1_JWB_ that are critical for its interaction with ZjCURT1A, we generated a series of site‐directed mutants based on a previous study (Aurin et al., 2020; Xue et al., 2024). The substituted amino acid sequences are illustrated in Figure 7a. Y2H assays showed that substitution of the residues at positions 27 and 37 in the first α‐helix (α1) both abolished the interaction with ZjCURT1A. Similarly, replacing leucine at position 75 in the second α‐helix (α2) with proline also disrupted binding to ZjCURT1A (Figure 7c). In contrast, the interaction remained unaffected when the residues at positions 46, 49, and 68 were mutated (Figure 7c). In summary, both α‐helices were indispensable, with residues 27 and 37 in α1 and leucine 75 in α2 being particularly critical for the PHYL1_JWB_–ZjCURT1A interaction. This finding is consistent with the key sites required for PHYL1_JWB_ to bind MADS‐box proteins, suggesting a conserved binding mechanism used by PHYL1_JWB_ to recruit different client proteins for degradation.

### The C‐terminal region containing a conserved thylakoid membrane curvature motif of ZjCURT1A, is required for binding to PHYL1_JWB_

**Figure 8:**
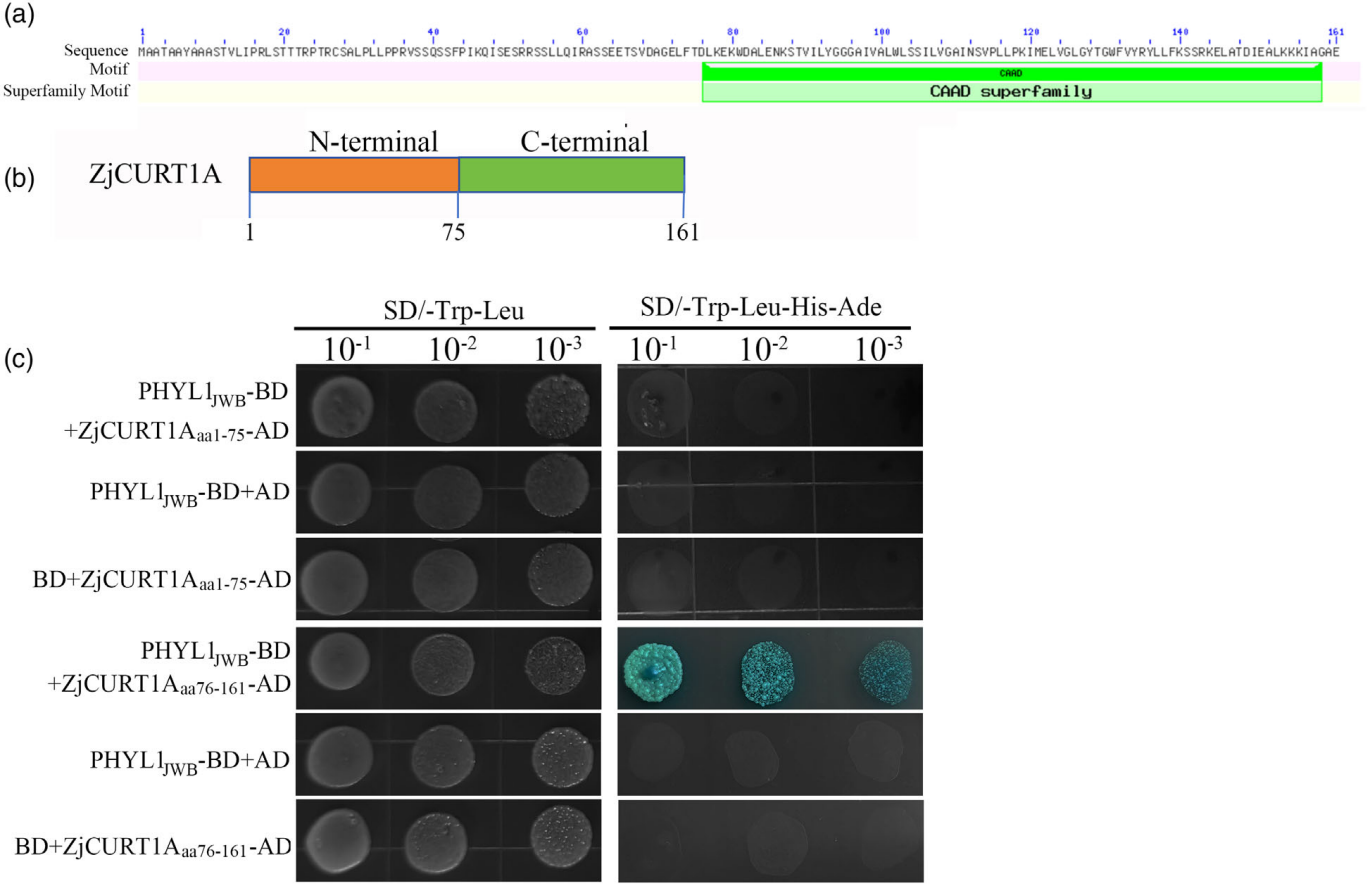
PHYL1_JWB_ interacts with the C‐terminal region of ZjCURT1A. (a) Schematic representation of the ZjCURT1A protein sequence, conserved motifs, and the conserved domain of the CURT1 superfamily. (b) The diagram in the box indicates the division of ZjCURT1A into N‐ and C‐terminal fragments, with numbers indicating amino acid positions. (c) Yeast two‐hybrid (Y2H) assays evaluated the interaction between PHYL1_JWB_ and the truncated ZjCURT1A mutants.

Based on the conserved functional domains of ZjCURT1A (the CURT1 family domain covering approximately amino acid residues 75–159), which includes known thylakoid membrane curvature‐related motifs, we divided ZjCURT1A protein into two segments: the N‐terminal (amino acid residues 1–75) and the C‐terminal (amino acid residues 76–161) regions (Figure 8a,b). Y2H assays were performed to map the interaction domain of ZjCURT1A with PHYL1_JWB_. The results showed that the N‐terminal segment alone was insufficient for binding PHYL1_JWB_, whereas the C‐terminal segment interacted strongly with PHYL1_JWB_ (Figure 8c), indicating that the interaction was mediated by the C‐terminal region, which contains the known thylakoid membrane curvature‐related motif CAAD (Cyanobacterial Aminoacyl‐tRNA synthetases Appended Domain) (Luque & Ochoa de Alda, 2014). Furthermore, binding assays between the C‐terminal segment of ZjCURT1A and a panel of PHYL1_JWB_ helix mutants confirmed the importance of the two α‐helical helixes (Figure S1), especially residues 27, 37 in α1 and Leu75 in α2, for the PHYL1_JWB_–ZjCURT1A interaction (Figure S2).

## DISCUSSION

Phytoplasma infection induces a range of typical symptoms in plants, commonly manifested as the ‘witches' broom’ phenotype. These symptoms include photosynthesis impairment, leaf yellowing (chlorosis), shoot proliferation (phyllody), greening of floral organs (virescence), sterility, and overall stunting (Huang et al., 2021; Xue et al., 2018, 2024). Although these symptoms have been documented for decades, the specific molecular mechanisms by which the pathogen remodels host development and physiology remain incompletely understood. The pathogenicity of phytoplasmas is largely attributed to their secreted effector proteins, which are delivered into host cells to manipulate key physiological processes. Hence, establishing direct causal links between individual effectors and specific symptom complexes has long been a central focus in the field. Previous studies have identified several phytoplasma effectors responsible for symptoms like dwarfing, witches' broom, and phyllody. For instance, the phytoplasma virulence factor TENGU, which suppresses auxin signaling by inhibiting the expression of Arabidopsis *ARF6* and *ARF8*, leading to dwarfism (Hoshi et al., 2009; Minato et al., 2014). The paulownia witches' broom phytoplasma effector PaWB‐SAP54 targets SPLs for ubiquitination and degradation via the 26S proteasome pathway, with the help of host RPN3, disrupting auxin, gibberellin, and cytokinin levels and causing witches' broom symptoms (Cao et al., 2021). The homologous effectors SAP54 and PHYL1_OY_ induce phyllody by targeting and degrading MADS‐domain transcription factors (Iwabuchi et al., 2019; MacLean et al., 2014). The JWB phytoplasma effectors SJP1 and SJP2 were identified to induce lateral bud outgrowth by disrupting ZjBRC1‐mediated auxin flux, and modulate floral transition and shoot branching by destabilizing the bifunctional regulator ZjTCP7 (Ma, Huang, et al., 2024; Ma, Zheng, et al., 2024). Zaofeng6 was found to promote shoot proliferation by downregulating *ZjTCP7* expression (Chen et al., 2022).

Among the various symptoms, photosynthesis impairment and the accompanying leaf yellowing represent a primary and economically significant outcome of infection. Previous research on phytoplasma‐induced photosynthesis impairment has primarily focused on correlative observations, such as reduced chlorophyll content and disruption of chloroplast ultrastructure (Liu et al., 2016; Xue et al., 2018). Broader hypotheses have implicated factors like nutrient competition, oxidative stress, or general toxins produced by the pathogen (Liu et al., 2016; Xue et al., 2018). However, effectors specifically and directly responsible for disrupting the photosynthetic apparatus have been notably scarce. This gap raises a fundamental question: is photosynthesis impairment merely a result of systemic, indirect stress responses, or is it driven by specific effector molecules that directly target components of the photosynthetic machinery?

**Figure 9:**
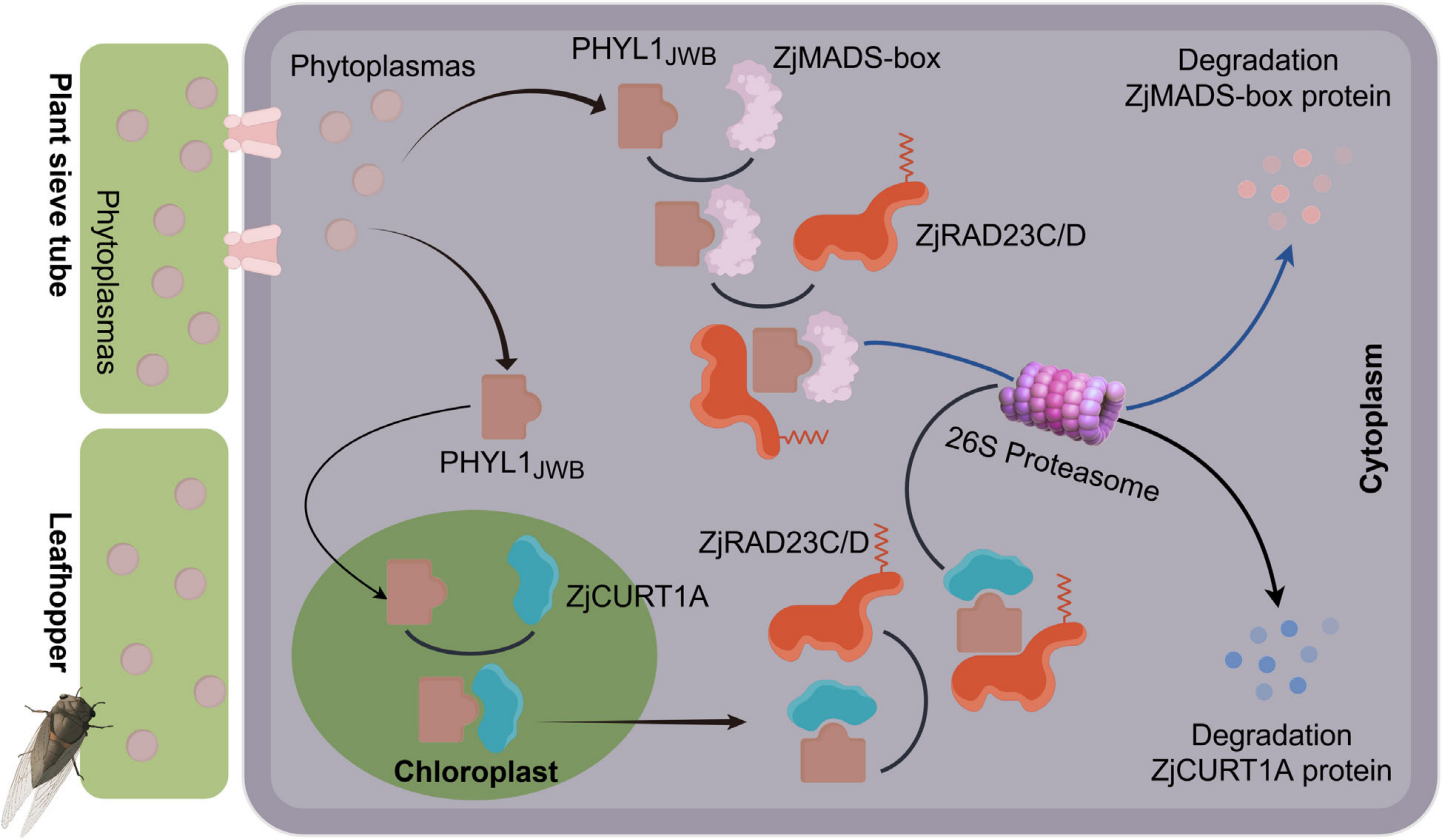
Model of PHYL1_JWB_‐Induced Abnormal Flower Development and Photosynthesis Impairment. During normal plant growth, MADS‐box transcription factors are maintained at appropriate levels to ensure proper floral organ development, while the thylakoid curvature protein ZjCURT1A is stably expressed to maintain normal chloroplast morphology and photosynthetic function. Upon feeding by phytoplasma‐infected leafhoppers, phytoplasmas enter the phloem sieve tubes of jujube trees and spread intercellularly. The secreted effector PHYL1_JWB_ targets distinct host proteins in a tissue‐specific manner. In floral primordia, it interacts with MADS‐box proteins; in leaf tissues, it binds to ZjCURT1A. Additionally, PHYL1_JWB_ interacts with the ubiquitin receptors RAD23C and RAD23D, and with their assistance, promotes the degradation of MADS‐box proteins and ZjCURT1A via the 26S proteasome in a ubiquitination‐independent manner, ultimately leading to abnormal flower formation and reduced chlorophyll content, impaired chlorophyll fluorescence kinetics, and overall photosynthetic dysfunction. The precise role of Rad23C/D in this process requires further investigation. Collectively, PHYL1_JWB_ exerts dual pathogenic effects—disrupting floral development and compromising photosynthesis—through a conserved mechanism involving ubiquitination‐independent proteasomal degradation of distinct host targets.

In this study, we addressed this gap by identifying and functionally characterizing the phytoplasma effector PHYL1_JWB_ as a key inducer of photosynthesis impairment. PHYL1_JWB_ is a phytopathogenic effector homologous to SAP54 and PHYL1_OY_, capable of inducing phyllody. This class of effectors are reported to interact with the ubiquitin receptors Rad23C and Rad23D and degrade floral development‐related MADS‐box protein family members, such as AP1, AGL6, SEP1‐4, and CAL, in a ubiquitination‐independent manner (Xue et al., 2024). In this study, we demonstrated that the phytoplasma effector PHYL1_JWB_ directly interacted with the integral thylakoid membrane protein ZjCURT1A and promoted its degradation via the 26S proteasome pathway. This degradation was likely analogous to that of floral MADS‐box transcription factors and may also depend on the ubiquitin receptors Rad23C and Rad23D (Figure 9). However, the precise role of Rad23C/D in this process warrants further investigation. Consequently, degradation of ZjCURT1A led to disruption of the thylakoid structure in chloroplasts and subsequent impairment of the photosynthetic system. This work provides direct mechanistic evidence rather than mere correlation. We demonstrated that overexpression of *PHYL1*_*JWB*_ alone, in both the native host (jujube) and the model plant *A. thaliana*, was sufficient to induce photosynthesis impairment. This included not only reduced chlorophyll content but also a specific decline in photosynthetic efficiency parameters (*F*_v_/*F*_m_, *F*_q_'/*F*_m_', ETR). Crucially, our ultrastructural analysis revealed that *PHYL1*_*JWB*_ directly induced severe disorganization of chloroplast ultrastructure, including the thylakoid membranes. These findings provide compelling evidence that JWB‐induced photosynthesis impairment is not a secondary side effect but a direct pathology initiated by a specific effector. This establishes *PHYL1*_*JWB*_ as a central player in manipulating the host's photosynthetic function, offering a novel, targeted molecular explanation for this classic disease symptom.

Previous studies have reported that PHYL1_JWB_ and its homologous proteins are predominantly localized to the nucleus and cytoplasm, where they regulate floral organ development (Kitazawa et al., 2022; Xue et al., 2024). In contrast, this study demonstrates that PHYL1_JWB_ is specifically targeted to the chloroplast and physically interacts with ZjCURT1A. This distinct subcellular localization reveals that PHYL1_JWB_ performs a functionally divergent role within the chloroplast, expanding the functional repertoire of the PHYL1 family beyond nuclear/cytoplasmic regulation. While direct experimental evidence for chloroplast localization of other PHYL1 homologs remains unavailable, the documented capacity of phytoplasma effectors to cross cellular and organellar membranes, including the chloroplast envelope (Ohtsu et al., 2024; Zhang et al., 2024), provides biologically plausible support for PHYL1_JWB_'s chloroplast targeting. Collectively, these findings reveal a previously uncharacterized subcellular localization and a chloroplast‐associated function for PHYL1_JWB_.

The PHYL1_JWB_ protein contains two α‐helical domains, a structure that is also conserved in other homologous proteins such as SAP54, PHYL1_OY_, and PHYL1_PnWB_ (Aurin et al., 2020; Iwabuchi et al., 2019, 2020; Liao et al., 2019). By constructing PHYL1_JWB_ mutants with deletions in different domains and amino acid substitutions, it was found that both two α‐helices of PHYL1_JWB_ were important for binding to MADS‐box proteins (Xue et al., 2024). Furthermore, consistent results from studies on its homologous proteins have shown that deletion of either helix in SAP54 abolishes its ability to bind to SEP3, AP1, or AGL6 (Aurin et al., 2020), and insertion of amino acids into either α‐helix of PHYL1_OY_ leads to loss of its phyllody‐inducing activity and ability to degrade floral MADS transcription factors (Iwabuchi et al., 2019). The second α‐helix of the peanut arbuscular phytoplasma effector PHYL1_PnWB_ is essential for its interaction with the K domain of SEP3 and degradation of it (Liao et al., 2019). In this study, we discovered that both α‐helices of PHYL1_JWB_ were necessary for binding to the integral thylakoid membrane protein ZjCURT1A. This indicates that the two α‐helixes are critical functional domains of this class of pathogenic effectors, whether mediating interactions with MADS‐box proteins leading to their degradation and the induction of phyllody symptoms, or with chloroplast thylakoid structural protein ZjCURT1A causing its degradation and impairment of photosynthesis. Further Y2H assays of amino acid substitution revealed that residues at positions 27 and 37 in the first α‐helix and the leucine at position 75 in the second α‐helix were particularly crucial for the interaction between PHYL1_JWB_ and ZjCURT1A. Previous studies have reported that these same residues (positions 27 and 37 in the first helix and leucine 75 in the second helix), are vital for PHYL1_JWB_'s interaction with its target MADS‐box proteins (Xue et al., 2024). These sites are also key in homologous proteins, for example, SAP54 requires L27, L65, and I69 for interaction with SEP3, AP1, or AGL6 (Aurin et al., 2020), while PHYL1_PnWB_ relies on I25, Y32, I60, and Y64 for binding to the K domain of SEP3 (Liao et al., 2019). These results collectively demonstrate the functional conservation of PHYL1‐like proteins in interacting with diverse target proteins.

Our findings expand the functional repertoire of PHYL1_JWB_ proteins in plants, revealing that in addition to inducing phyllody, they also impair photosynthesis by disrupting chloroplast ultrastructure, thereby contributing to abnormal plant growth. These insights provide a deeper understanding of the mechanistic diversity of PHYL1‐like effectors in plant–pathogen interactions. It will be of particular interest in future studies to explore whether other PHYL‐like proteins similarly target the photosynthetic apparatus in different host plants. Notably, our study highlights the functional pleiotropy of pathogenic effectors, suggesting that investigations into their roles, particularly in the context of phytoplasma pathogenicity, should consider potential impacts on multiple physiological processes.

## MATERIALS AND METHODS

### Plant materials

Healthy and JWB‐infected jujube trees cultivated at the Fuping Jujube Comprehensive Test Station of Hebei Agricultural University, China, were used as the source of leaf materials for experiments. Tissue‐cultured plantlets of both healthy and JWB‐infected jujube were maintained in a growth chamber under controlled conditions with a 16‐h light/8‐h dark photoperiod.

Seeds of sour jujube (*Ziziphus jujuba* var. *spinosa* (Bunge) Hu) were germinated on moistened gauze in Petri dishes within a constant‐temperature incubator set at 28°C under a 16‐h light/8‐h dark cycle for 2–3 days. Seedlings with embryonic roots approximately 1 cm in length were carefully transplanted into soil and grown until cotyledon expansion was fully achieved.

*Arabidopsis thaliana* seeds were surface‐sterilized using standard protocols and sown on ½ Murashige and Skoog (MS) medium supplemented with 0.8% agar. The seeded plates were subjected to stratification at 4°C for 2 days to synchronize germination, then transferred to a growth chamber maintained at 22°C with a 16‐h light/8‐h dark cycle. The Columbia‐0 (Col‐0) ecotype was used as WT control.

### Determination of chlorophyll content

The chlorophyll content in jujube leaves was determined using the 95% ethanol extraction method (Knudson et al., 1977). Fresh leaf samples (approximately 100 mg) were collected, cut into small strips, and immersed in 20 ml of 95% ethanol for pigment extraction under dark conditions. Extraction was carried out until the leaf tissues became colorless, indicating complete chlorophyll solubilization. An aliquot of the resulting extract was transferred to a cuvette, and absorbance was measured at 665 and 649 nm using a UV–visible spectrophotometer. All measurements were performed with three biological replicates to ensure data reliability.

Chlorophyll concentrations were calculated using the following equations (Riccardi et al., 2014):

$$\mathrm{Chl}\;a=13.95\times {\mathrm{OD}}_{665}-6.88\times {\mathrm{OD}}_{649}.$$

$$\mathrm{Chl}\;b=24.96\times {\mathrm{OD}}_{649}-7.32\times {\mathrm{OD}}_{665}.$$

$$\text{Total}\;\mathrm{Chl}\;\text{content}\left(\mathrm{mg}\;g^{-1}\right)=\begin{array}{l} {}[\left(\mathrm{Chl}\;a+\mathrm{Chl}\;b\right)\\ \times \text{extraction volume}\\ \times \text{dilution factor}]/\text{leaf fresh weight.} \end{array}$$

### Measurement of chlorophyll fluorescence kinetic parameters

Fresh leaf samples were placed on the detection plate of a PlantExplorerPro+ multifunctional plant photosynthesis imaging system, and the appropriate data modules were selected based on the parameters to be measured. Prior to measurement, the leaf samples were dark‐adapted for 30 min to ensure that the reaction centers of PSII were fully open and the electron transport chain was in an oxidized state. Upon sudden exposure to visible light, the leaf samples emitted a dark‐red fluorescence signal of varying intensity, which initially increased and then decreased. The initial value of this fluorescence signal was recorded as the initial fluorescence (*F*_o_), and the maximum value as the maximum fluorescence (*F*_m_). The maximum photochemical efficiency of PSII was calculated as *F*_v_/*F*_m_ = (*F*_m_ − *F*_o_)/*F*_m_.

Subsequently, an actinic light was turned on to simulate the ambient light environment of the plants. After the fluorescence signal decreased to a stable level, the steady‐state fluorescence (*F*_s_') was recorded. A saturating light pulse was then applied again to determine the maximum fluorescence under light (*F*_m_'), allowing for the calculation of the actual photochemical efficiency of PSII as *F*_q_'/*F*_m_' = (*F*_m_' – *F*_s_')/*F*_m_', as well as the ETR.

### TEM observation of chloroplast ultrastructure in leaves

The sample preparation procedure was conducted as follows: Young leaves were collected from corresponding plants and cut into segments approximately 1 mm in length. The leaf segments were immediately immersed in 4% glutaraldehyde, subjected to vacuum infiltration for 30 min, and then stored overnight at 4°C. Subsequently, the samples were rinsed three times with 0.1 m phosphate buffer to remove residual fixative. Post‐fixation was performed using 0.1% osmium tetroxide under vacuum for 2.5 h. After post‐fixation, the samples were washed three times with 0.1 m phosphate buffer, each wash lasting 10 min. Dehydration was carried out through a graded acetone series, followed by embedding in epoxy resin and polymerization in an oven at 37°C for 48 h. Ultrathin sections (60–70 nm thick) were prepared using an ultramicrotome, collected onto uncoated copper grids, and double‐stained with uranyl acetate and lead citrate. The sections were examined under a transmission electron microscope (Hitachi H‐7650).

### Generation of *PHYL1*_*JWB*_ and *ZjCURT1A* overexpression lines in sour jujube seedlings via transient transformation

The full‐length coding sequences of *PHYL1*_*JWB*_ and *ZjCURT1A* were cloned into the *pCAMBIA1300‐GFP* overexpression vector. The resulting constructs were then transformed into *Agrobacterium tumefaciens* strain GV3101 (pMP90). Bacterial suspensions carrying the respective plasmids were injected into the cotyledons of sour jujube seedlings following a previously established method (Yang et al., 2025). Seedlings injected with the empty vector served as the negative control. After two times of infection, leaves and branches were harvested for RNA extraction and subsequent quantitative reverse transcriptase‐polymerase chain reaction (qRT‐PCR) analysis to verify gene overexpression. The confirmed overexpression lines were used for further assays. The primer sequences used in this assay are provided in Table S1.

### Generation of *PHYL1*_*JWB*_ overexpression lines of *A. thaliana*

The construct of 35S:*PHYL1*_*JWB*_ *‐GFP* was introduced into Col‐0 ecotype of *A. thaliana* via *A. tumefaciens*‐mediated floral dip transformation. Since constitutive overexpression of *PHYL1*_*JWB*_ resulted in sterile plants, heterozygous T1 transgenic lines were selected and used for subsequent experiments.

### Yeast two‐hybrid assays

The full‐length coding sequence of *PHYL1*_*JWB*_, including its secretory signal peptide, was cloned and inserted into the *pGBKT7* vector. Using a cDNA library from ‘Dongzao’ jujube fruit constructed in the *pGADT7* vector as the prey, a Y2H screen was performed to identify proteins interacting with PHYL1_JWB_. The coding sequences of PHYL1_JWB_ and its mutant proteins were cloned into the *pGBKT7* vector, while the full‐length coding sequence of the candidate protein ZjCURT1A and its mutant proteins were cloned into the *pGADT7* vector. Different pairs of these constructs were co‐transformed into the yeast strain *Saccharomyces cerevisiae* AH109. Successful transformants were selected on synthetic complete medium lacking tryptophan and leucine (SD/‐Trp‐Leu), while protein–protein interactions were assessed on synthetic complete medium lacking tryptophan, leucine, adenine, and histidine (SD/‐Trp‐Leu‐Ade‐His). The yeast plates were incubated at 28°C for 5 days prior to observation and imaging. The primers used in this assay are provided in Table S1.

### Protein expression and purification

The full‐length coding sequence of *ZjCURT1A* was amplified by PCR and cloned into the *pGEX‐4T‐2* vector (Amersham Bio‐sciences, Piscataway, NJ, USA) to generate glutathione S‐transferase (GST)‐tagged recombinant GST‐ZjCURT1A protein. The full‐length coding sequence of PHYL1_JWB_ was cloned into the *pMAL‐c2* vector for the maltose‐binding protein (MBP)‐tagged fusion protein MBP‐PHYL1_JWB_. All primers employed for constructing these recombinant plasmids are provided in Table S1.

The resulting plasmid constructs were individually transformed into *Escherichia coli* strain BL21 (DE3) for protein expression. Recombinant protein production was induced by the addition of isopropyl β‐D‐1‐thiogalactopyranoside (IPTG). Following induction, the corresponding GST‐ or MBP‐tagged proteins were affinity‐purified following the manufacturer's instructions (GE Healthcare, Chicago, IL, USA). The concentration of each purified protein was quantified using the Protein Assay Kit II (Bio‐Rad, Hercules, CA, USA).

### *In vitro* pull‐down assay

In the GST pull‐down assay to examine the PHYL1_JWB_–ZjCURT1A interaction, 10 μg of either GST‐ZjCURT1A or GST control protein was first prebound to glutathione‐Sepharose 4B resin (Amersham Pharmacia, Chalfont St Giles, UK) in 200 μl of PBS buffer (10 mM Na_2_HPO_4_, 1.8 mM KH_2_PO_4_, 140 mM NaCl, 2.7 mM KCl, pH 7.4) for 2 h at 4°C. Following this, 2 μg of purified MBP‐PHYL1_JWB_ was added and the mixture was incubated for another 2 h at 4°C. After incubation, the resin was washed three times with PBS. Bound proteins were eluted and separated by 10% SDS‐PAGE, followed by immunoblotting analysis. MBP‐tagged proteins were probed with an anti‐MBP antibody (Abclonal), while GST fusion proteins were detected using an anti‐GST antibody (Abclonal, Woburn, MA, USA).

### Firefly luciferase complementation imaging assay

The coding sequence of *ZjCRUT1A* was inserted into the 35S:cLuc vector, while *PHYL1*_*JWB*_ was cloned into the 35S:nLuc vector for split‐luciferase complementation assays. These constructs were transformed into *A. tumefaciens* strain GV3101 (pMP90) and subsequently co‐infiltrated into leaves of *Nicotiana benthamiana*. LCI assays were conducted following a previously described protocol (Chen et al., 2008). All primers used for plasmid construction are listed in Table S1.

### Co‐immunoprecipitation assay

To generate the construct *35S:ZjCURT1A‐MYC*, the full‐length coding sequence of *ZjCURT1A* was amplified by PCR and inserted into the *pSuper1300‐MYC* vector. Similarly, the coding sequence of *PHYL1*_*JWB*_ was cloned into the *pCAMBIA1300‐GFP* vector to produce *35S:PHYL1*_*JWB*_ *‐GFP*. The plasmid pairs *35S:GFP* + *35S:ZjCURT1A‐MYC* and *35S:PHYL1*_*JWB*_ *‐GFP* + *35S:ZjCURT1A‐MYC* were separately co‐expressed in *N. benthamiana* leaves via agroinfiltration.

Total protein was extracted using a Plant Protein Extraction Kit (CWBIO, Jiangsu, China; Cat# CW0885M). The lysate was incubated with GFP‐Nanoab‐Magnetic beads (LABLEAD, Beijing, China; Cat# GNM‐125‐5K). Proteins bound to the beads were separated by SDS‐PAGE and analyzed by immunoblotting. ZjCURT1A‐MYC was detected with an anti‐MYC antibody (CWBIO; Cat# CW0299M), while GFP and GFP‐tagged fusion proteins were probed with an anti‐GFP antibody (Cat# AE012; ABclonal).

### Bimolecular fluorescence complementation assay

For BiFC analysis, *PHYL1*_*JWB*_ was fused to the C‐terminal fragment of yellow fluorescent protein (cYFP) in the *pSPYCE(M)* vector, while *ZjCRUT1A* was linked to the N‐terminal fragment of YFP (nYFP) using the *pSPYNE(R)173* vector, following an established protocol (Waadt et al., 2008). The resulting constructs were transiently co‐expressed in *N. benthamiana* leaves. Fluorescence signals were visualized with a confocal laser‐scanning microscope (LSM 910; Carl Zeiss, Oberkochen, Baden‐Württemberg, Germany). All primers used for plasmid construction are provided in Table S1.

### Virus‐induced gene silencing of *ZjCURT1A* in sour jujube seedlings

VIGS was employed to silence *ZjCURT1A* in sour jujube seedlings following a previously established method (Zhang et al., 2023). Briefly, a 200‐bp specific fragment of *ZjCURT1A* was cloned into the *pTRV2* vector to generate *pTRV2‐ZjCURT1A*. *Agrobacterium tumefaciens* strain GV3101 carrying either *pTRV2‐ZjCURT1A* + *pTRV1* (silencing construct) or *pTRV2*‐empty + *pTRV1* (negative control) was infiltrated into the cotyledons of sour jujube seedlings. After two rounds of infiltration, leaves and branches were collected for RNA extraction and subsequent qRT‐PCR analysis to confirm gene silencing. The validated *ZjCURT1A*‐silenced lines were used for further functional assays. The primers used in this assay are provided in Table S1.

### Assay for PHYL1_JWB_ mediated degradation of ZjCURT1A protein

To investigate whether PHYL1_JWB_ induces the degradation of ZjCURT1A, the plasmid pairs 35S:GFP + 35S:ZjCURT1A‐MYC and 35S:PHYL1_JWB_‐GFP + 35S:ZjCURT1A‐MYC were separately co‐expressed in *N. benthamiana* leaves via agroinfiltration. For proteasome inhibition, leaves expressing the proteins of interest were sprayed daily with 20 μM MG132 (Sigma, Burlington, MA, USA) prior to sampling. An equivalent volume of DMSO was applied as a mock control. Three days post‐infiltration, leaf tissues were harvested and total proteins were extracted using an extraction buffer. Protein samples were then separated by SDS‐PAGE and analyzed by Western blotting to assess ZjCURT1A‐MYC levels. Band intensities were measured using ImageJ software and normalized against the β‐actin loading control.

### Statistical analysis

Data analysis was conducted using Student's *t*‐test with SPSS 27 software. Statistical significance was defined as *P < 0.05, **P < 0.01, and ***P < 0.001. Values represent mean ± SD from three independent replicates. All graphs were created using GraphPad Prism 10.

## AUTHOR CONTRIBUTIONS

ZL, XZ, ML and JZ conceived and designed the experiments. FH, CA, CY, and JF, and MY performed the experiments and analyzed the data. CX and HL were responsible for all vector constructions of PHYL1_JWB_ mutants used in Y2H assays. LD, JC and XQ performed sample cultivation and collection. ZL and XZ wrote the manuscript.

## CONFLICT OF INTEREST

The authors declare that they have no known competing financial interests or personal relationships that could have appeared to influence the work reported in this paper.

## Supporting information


**Figure S1.** Y2H assays showing that the two α‐helices of PHYL1_JWB_ were the key domains mediating its interaction with the C‐terminal region of ZjCURT1A.
**Figure S2.** Y2H assays showing that the residues 27, 37 in α1 and Leu75 in α2 of PHYL1_JWB_ were key for the PHYL1_JWB_–ZjCURT1A interaction.
**Table S1.** Detailed information of primers used in study.

## Data Availability

Data sharing is not applicable to this article as no datasets were generated or analyzed during the current study.
