# Supplementary material for: The pathogenic effector PHYL1JWB interacts with ZjCURT1A to mediate jujube witches' broom‐induced photosynthesis impairment of jujube trees
Source: Plant J. 2026 Sep 7;127(5):e71104. doi: 10.1111/tpj.71104 (PMC13549705; doi:10.1111/tpj.71104)
Supplement: Supplementary file 1 [file TPJ-127-0-s001.doc]

**Supporting Information**

**The Pathogenic Effector PHYL1_JWB_ Interacts with ZjCURT1A to Mediate Jujube Witches' Broom-Induced Photosynthesis Impairment of Jujube Trees**

**Fangfang Huang^a, #^, Changfeng Ai^a, #^, Chong Yang^b, #^, Jie Feng^a, #^, Meng Yang^a, #^, Li Dai^a, e^, Chaoling Xue^c^, Hongtai Li^d^, Jiaxue Cui^a^, Xiangjie Qu^a^, Jin Zhao^d^, Mengjun Liu^a, e, *^, Xuan Zhao^a, e, *^ and Zhiguo Liu^a, e, *^**

^a^College of Horticulture, Hebei Agricultural University, Baoding Hebei, China

^b^National Engineering Research Center for Agriculture in Northern Mountainous Areas, Hebei Agricultural University, Baoding, Hebei, China

^c^Shanxi Sericulture Science Research Institute, Shanxi Agricultural University, Yuncheng, Shanxi, China

^d^College of Life Science, Hebei Agricultural University, Baoding, Hebei, China

^e^Research Center of Chinese Jujube, Hebei Agricultural University, Baoding Hebei, China

*For correspondence: e-mail jujubeliu@[163.com](mailto:zhaoxuanzn@163.com) (Z. Liu); [zhaoxuanzn@163.com](mailto:zhaoxuanzn@163.com) (X. Zhao); kjliu@hebau.edu.cn (M. Liu);

^#^These authors contributed equally to this work.

***Supplemental Table Detailed information of primers used in study***

| **Primer name** | **Primer sequence（5‘-3’）** |
| --- | --- |
| *35S:PHYL1_JWB_-GFP*-F | ACGAGCTCGGGTACCATGATGCAAATAAAAAATAAAT |
| *35S:PHYL1_JWB_-GFP*-R | GACTCTAGAGGATCCGTTATTTTCATCATTTAAATTTT |
| *35S:ZjCURT1A-GFP*-F | CGGGGACTCTTGACCATGGGATGGCAGCAACGGCG |
| *35S:ZjCURT1A-GFP*-R | CTAGTCAGATCTACCATGGG CTCGGCTCCGGCAATCT |
| *ZjCURT1A-pRTV2*-F | GGTTACCGAATTCTCTAGAGCAATTTCAGAGTCCCGCAGGTCT |
| *ZjCURT1A-pRTV2*-R | GAGACGCGTGAGCTCGGTACCCGCTAGTTCTTTTCTGCTTGACTTGA |
| *pGADT7-ZjCURT1A*-F | ATGGAGGCCAGTGAATTCATGGCAGCAACGGCG |
| *pGADT7-ZjCURT1A*-R | ATGCCCACCCGGGTGGAATTCCTCGGCTCCGGCAAT |
| *pGBKT7-PHYL1_JWB_*-F | GCCATGGAGGCCGAATTCATGATGCAAATAAAAAATAAAT |
| *pGBKT7-PHYL1_JWB_*-R | ACGGATCCCCGGGAATTCGTTATTTTCATCATTTAAATTTT |
| *ZjCURT1A-GST*-F | CTGGTTCCGCGTGGATCCATGGCAGCAACGGCGG |
| *ZjCURT1A-GST*-R | GGGAATTCCTGGGGATCCCTCGGCTCCGGCAATCTT |
| *PHYL1_JWB_-MBP*-F | GAGGGAAGGATTTCACATATGATGATGCAAATAAAAAATAAAT |
| *PHYL1_JWB_-MBP*-R | CTTTCGTTTTATTTGAAGCTTGTTATTTTCATCATTTAAATTTT |
| *ZjCURT1A-MYC*-F | CTGCAGGGGCCCGGGGTCGACATGGCAGCAACGGCG |
| *ZjCURT1A-MYC*-R | GAGCTTTTGCTCCATGGTACCCTCGGCTCCGGCAAT |
| *ZjCURT1A(K->R)-MYC*-F | CTGCAGGGGCCCGGGGTCGACATGGCAGCAACGGCGGCGTAT |
| *ZjCURT1A(K->R)-MYC*-R | GAGCTTTTGCTCCATGGTACCCTCGGCTCCGGCAATCCTCCT |
| *ZjCURT1A-cLuc*-F | gcgtcccggggcGGTACCATGGCAGCAACGGCG |
| *ZjCURT1A-cLuc*-R | aaagctctgcagGTCGACCTCGGCTCCGGCAA |
| *PHYL1_JWB_-nLuc*-F | GGGGACGAGCTCGGTACCATGATGCAAATAAAAAATAAAT |
| *PHYL1_JWB_-nLuc*-R | ATCGTATGGGTAGTCGACGTTATTTTCATCATTTAAATTTT |
| *ZjCURT1A-nYFP*-F | ACGGGGGACGAGCTCATGGCAGCAACGGCGG |
| *ZjCURT1A-nYFP*-R | ATGGAGCAAGTCGACCTCGGCTCCGGCAATCTT |
| *PHYL1_JWB_-cYFP*-F | ACGGGGGACGAGCTCATGATGCAAATAAAAAATAAAT |
| *PHYL1_JWB_-cYFP*-R | ATGTACCCAGTCGACGTTATTTTCATCATTTAAATTTT |
| *ZjCURT1A-_aa1-75_-AD*-F | ATGGAGGCCAGTGAATTCATGGCAGCAACGGCG |
| *ZjCURT1A-_aa1-75_-AD*-R | ATGGAGGCCAGTGAATTCATCTGTGAAAAGCTCTCCAGC |
| *ZjCURT1A-_aa76-161_-AD*-F | ATGGAGGCCAGTGAATTCTTGAAGGAGAAGTGGGATGC |
| *ZjCURT1A-_aa76-161_-AD*-R | ATGCCCACCCGGGTGGAATTCCTCGGCTCCGGCAAT |

**Supplemental Figure S1.** Y2H assays showing that the two α-helices of PHYL1_JWB_ were the key domains mediating its interaction with the C-terminal region of ZjCURT1A.

**Supplemental Figure S2.** Y2H assays showing that the residues 27, 37 in α1 and Leu75 in α2 of PHYL1_JWB_ were key for the PHYL1_JWB_–ZjCURT1A interaction.
